# Use of thermal imaging in the detection of the diabetic foot- review

**DOI:** 10.1007/s11154-025-09999-w

**Published:** 2025-10-14

**Authors:** Aleksandra Mrowiec, Teresa Kasprzyk-Kucewicz, Daria Wziątek-Kuczmik, Agata Stanek, Armand Cholewka

**Affiliations:** 1 Faculty of Science and Technology, University of Silesia, Katowice, 40-007 Poland; 2 Department of Cranio-Maxillofacial Surgery, Faculty of Medical Sciences, Medical University of Silesia, Katowice, 40-027 Poland; 3 Department of Internal Medicine, Metabolic Diseases, and Angiology, Faculty of Health Sciences in Katowice, Medical University of Silesia, Ziołowa 45-47 St, Katowice, 40- 635 Poland; 4 Upper-Silesian Medical Centre of the Medical University of Silesia in Katowice, Ziołowa 45-47 St, Katowice, 40- 635 Poland

**Keywords:** Thermal imaging, Diabetic foot ulcers, Diagnosis, Diabetes mellitus

## Abstract

Diabetic foot ulcers (DFUs) are among the most serious complications of diabetes mellitus, often resulting in infection, amputation, and increased mortality. Early detection is essential but remains difficult due to the complex interaction of neuropathy, vascular disease, and immune dysfunction. This review examines the effectiveness of thermal imaging, including approaches supported by artificial intelligence (AI), as a non-invasive tool for identifying early signs of DFUs. A total of 49 studies published between 1991 and 2024 were analysed, focusing on adult patients and primary research only. Findings show that thermal imaging can detect abnormal skin temperature patterns and early inflammation, key indicators of DFU development. AI techniques, such as machine learning and neural networks, further enhance diagnostic accuracy by identifying subtle patterns and predicting ulcer risk. Despite promising results, several limitations were noted: lack of standardised imaging protocols, inconsistent equipment quality, and small sample sizes in many studies. To improve clinical reliability, future work should focus on developing standard procedures, integrating AI with high-resolution thermal cameras, and validating these systems in real-world hospital and home-care settings. Overall, thermal imaging, especially when combined with AI, shows strong potential as a practical, non-invasive method for early DFU detection and monitoring.

## Introduction

Diabetes mellitus remains one of the most prevalent non-communicable diseases worldwide. According to the International Diabetes Federation, by 2045, an estimated 700 million individuals will live with diabetes [1]. Furthermore, the prevalence of diabetes among adults has risen markedly, from 4.7% in 1980 to 8.5% in 2016, as reported by the World Health Organization (WHO) [2].

Among the serious chronic complications associated with diabetes, diabetic foot disease (DFD) represents a significant clinical and economic burden. DFD encompasses a spectrum of pathological conditions, primarily diabetic foot ulcers (DFU), which arise due to peripheral neuropathy, peripheral arterial disease (PAD), and impaired wound healing [3–5]. The estimated global incidence of DFU is approximately 6% [4], and up to 20% of affected patients may require limb amputation as a life-saving intervention [5].

Neurological dysfunction is a central contributor to DFD. Peripheral neuropathy gradually loses protective sensation, while motor neuropathy results in muscular atrophy and anatomical foot deformities that generate abnormal pressure points, contributing to skin breakdown and ulcer formation [6–8]. Autonomic neuropathy further compromises the skin’s integrity by reducing sweat production, predisposing to cracking and infection. Early symptoms may include pain, tingling, or hypersensitivity, progressing to numbness, weakness, imbalance, and instability [6, 7, 9].

DFU development is often compounded by vascular insufficiency. Ischemia caused by PAD is implicated in nearly 90% of diabetic foot amputations [10, 11]. Chronic microvascular inflammation contributes to capillary basement membrane thickening, impeding nutrient and leukocyte delivery to tissues. Compromised vasodilation in response to local inflammation further exacerbates ischemic injury.

Moreover, impaired immune function in diabetic patients hinders ulcer healing. Hyperglycemia negatively impacts leukocyte chemotaxis, phagocytosis, and bactericidal activity [12–14]. Biofilm formation at ulcer sites delays healing and fosters recurrent infections. Molecular mechanisms such as overexpression of c-Myc and β-catenin, and increased apoptosis of T lymphocytes, have also been associated with poor wound resolution in DFU [12, 15].

Diagnosis of DFD requires a multidisciplinary approach, including detailed assessment of neurological, vascular, musculoskeletal, and dermatological parameters [16–18]. Imaging modalities such as X-ray, MRI, and ultrasound may be employed, depending on clinical presentation and the need for differential diagnosis.

Although various classification systems are used to evaluate DFU—such as the Meggitt-Wagner, University of Texas, SINBAD, and WIfI systems—they often focus on wound size, depth, and presence of ischemia or infection, while failing to account for the full complexity and dynamic progression of the disease [19–25]. Many tools do not sufficiently address subclinical changes or facilitate early detection before ulcer formation.

Given these limitations, there is a growing interest in non-invasive, objective diagnostic methods that can enhance early screening of diabetic foot complications. Thermal imaging emerges as a promising modality in this regard. It enables rapid, contactless measurement of skin surface temperature, identifying localized temperature changes that may signal underlying inflammation or perfusion abnormalities—markers of potential ulceration [26].

Recent evidence supports the clinical utility of thermal imaging in monitoring DFU. For instance, Glik et al. demonstrated improved outcomes when thermal imaging was combined with hyperbaric oxygen therapy, contributing to faster healing and wound size reduction [27]. Gebala-Prajsnar et al. further highlighted the role of adjunct physical medicine interventions in reducing amputation risk [28]. Additionally, vascular assessment remains essential, as the presence of lower extremity artery disease (LEAD) significantly influences prognosis and healing, warranting tailored strategies in diabetic populations [29].

While thermal imaging shows potential, its routine clinical use in early DFD screening remains underexplored. A search of the PubMed database using the term “thermal imaging of the diabetic foot” identified 80 relevant publications between 1981 and 2024, including seven in the most recent year.

This review aims to evaluate the potential role of thermal imaging as a non-invasive tool for early detection of diabetic foot complications, including pre-ulcerative conditions, in patients with type II diabetes. In doing so, it seeks to synthesize current evidence, identify gaps in knowledge, and propose directions for future clinical integration.

## Materials and methods

### Aim and research question

The review aims to explore the use of thermography to detect DFU in patients with different diabetes mellitus levels. The research question was: “Is thermal imaging useful in detecting the early stages of DFU development?”.

Based on the collected articles, an assessment was made regarding the usefulness of thermal imaging in detecting pre-ulcerative conditions and evaluating subsequent complications, i.e., ulceration.

## Search strategy

In searching for scientific publications for this paper, the search for the term „thermal imaging of the diabetic foot” was used. Databases searched: PubMed, Google Scholar, and ScienceDirect. Restrictions were applied regarding the publication of articles, considering the range of years from 1991 to 2024. Diagnosis of adult patients, i.e., patients aged > 18 years, was considered.

Articles include A short Report, The Original Research (The Study), A Case Study, A Pilot Study, a Regular Article, a Feasibility Study, and a Brief Original Article.

Inclusion criteria were all quantitative, primary human studies written in English. Studies focusing on the early detection of DFU were included.

Review papers, conference proceedings, opinion papers, and those involving participants aged < 18 years were excluded.

**Fig. 1:**
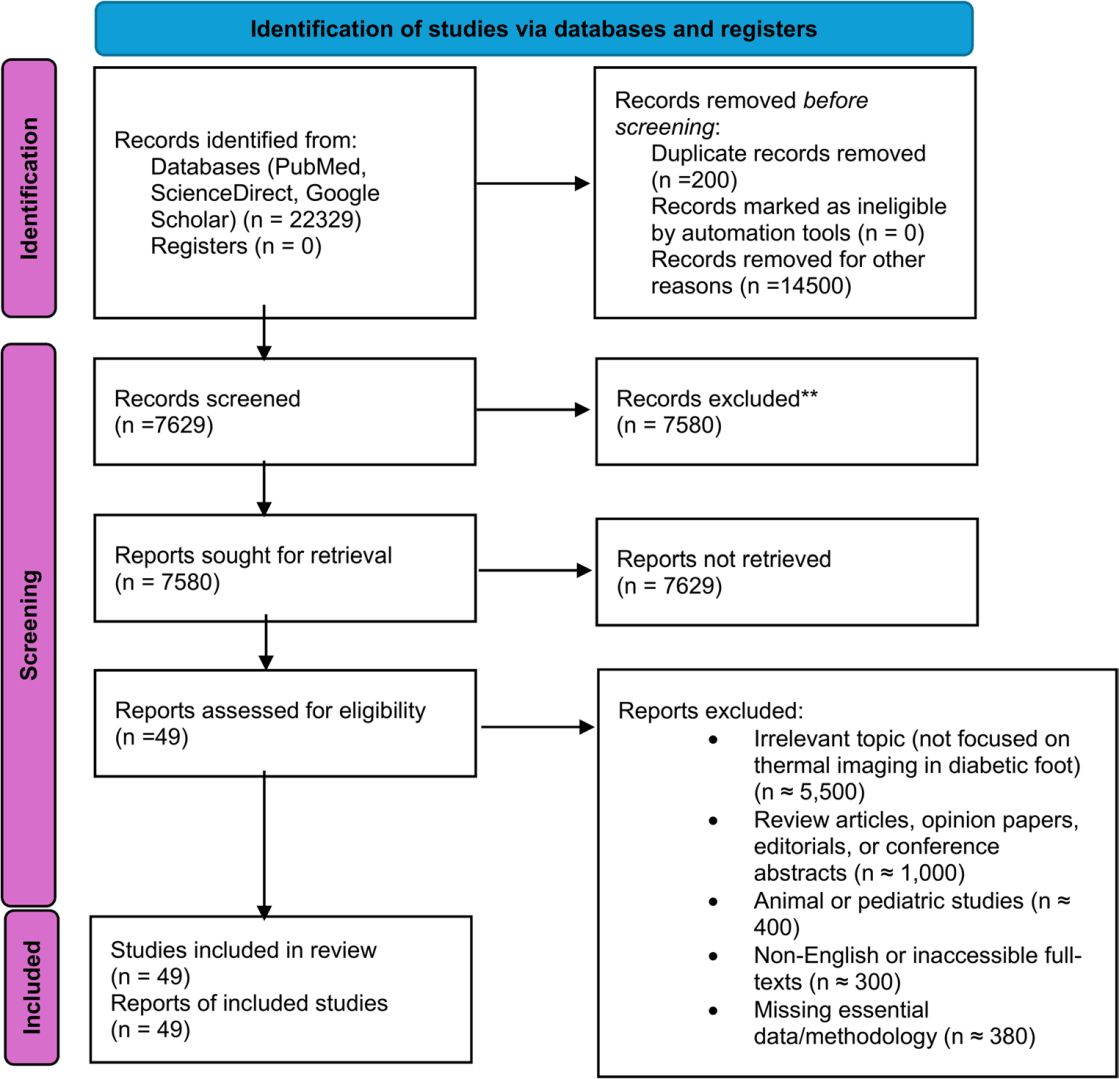
PRISMA flow diagram for study selection, where n – indicates the number of papers relevant to each step

The review protocol was conducted according to the recommendations of the PRISMA-P (Preferred Reporting Items for Review and Meta-Analysis Protocols) 2015 checklist [30]. The Identification of studies via databases and registers is shown in Fig. 1. As can be seen, from more than 18.080 studies, only 49 reports were evaluated for eligibility due to exclusion criteria such as lack of detailed patient data, Outdated results, or not applicable to the subject, etc. The review protocol was registered on PROSPERO (registration ID CRD420251036340).

Thus, forty-nine studies were finally included and formed the basis of this review [31–79].

During the initial screening of 22,329 records retrieved from PubMed, ScienceDirect, and Google Scholar, a large proportion was excluded based on title and abstract due to irrelevance to the topic, duplication, non-primary research design (e.g., reviews, editorials), or language/population mismatch. Google Scholar results often included poorly indexed content with limited clinical value. After full-text review, 49 studies met the eligibility criteria and were included in the final analysis.

## Result

Forty-nine articles were selected based on a PubMed, Google Scholar, and ScienceDirect search for the term ‘thermal imaging of the diabetic foot’. The following table contains basic information about the selected articles: Title, Author, Year, Type, Sample, Objectives, Results, and Conclusions. The articles have also been sorted by year (Table 1).

Thermal imaging demonstrated consistent potential across the 49 included studies in detecting early signs of diabetic foot complications, particularly when integrated with artificial intelligence techniques. Most studies confirmed that thermographic analysis revealed localized temperature differences of ≥2 °C in ulcer-prone regions. Several machine learning models (e.g., DFTNet, AdaBoost+RF, EfficientNetB0) achieved high classification accuracy (>90%) in identifying ulceration risk, even when using mobile or low-cost devices. However, methodological limitations were common, including small sample sizes, lack of standard protocols, and heterogeneous imaging conditions. Deep learning studies generally showed better sensitivity and specificity than classical statistical models. Overall, the findings support the utility of thermal imaging, especially in combination with AI, as a non-invasive screening tool for early diabetic foot ulcer detection. However, further validation in real-world clinical settings is needed.

**Table 1:** Summary table of DFU identification using thermal imaging

Lp.	Title	Author	Year	Type	Sample	Objectives	Results	Conclusions
1	Contact Thermography of Painful Diabetic Neuropathic Foot [31]	Chan AW, MacFarlane IA, Bowsher DR	1991	The Short report	68 participants (33 in the control group and 35 with diabetes)	Investigated regional differences in skin blood flow (measured by contact thermography) in the diabetic neuropathic foot.	In thermograms of diabetic patients with PPN and control subjects, the diabetic patients had significantly higher MFT and less consistent temperature patterns. There was no correlation between MFT and pain scores, and a slight decrease in MFT was observed over time, with no change in pain scores.	Patients with DM and PPN show increased skin temperature, particularly in areas exposed to stress. However, foot temperature does not correlate with neuropathic pain severity, suggesting that elevated temperature may indicate areas at risk of ulceration rather than being directly related to pain.
2	Preventing Diabetic Foot Ulcer Recurrence In High-Risk Patients [32]	Lavery LA, Higgins KR, Lanctot DR, et al.	2007	The Study	173 participants (with a previous history of diabetic foot ulceration were assigned to standard therapy, structured foot examination, or enhanced therapy groups)	Assess the effectiveness of a temperature monitoring instrument in reducing the incidence of foot ulcers among high-risk individuals with diabetes.	Enhancing therapy with a thermal imaging camera significantly reduced the risk of foot ulceration in diabetic patients compared to standard treatment and structured foot examination, with better adherence to temperature monitoring vital to preventing ulceration.	Providing patients with high-risk diabetes with simple at-home temperature monitoring devices significantly reduces the risk of foot ulceration, allowing activity levels to be adjusted promptly and proving more effective than traditional self-monitoring methods.
3	Correlation between plantar foot temperature and diabetic neuropathy: a case study by using an infrared thermal imaging technique [33]	Bagavathiappan S, Philip J, Jayakumar T, et al.	2010	A case study	112 participants with diabetes	Investigation of the relationship between sole temperature and diabetic neuropathy in patients with type 2 diabetes using non-invasive infrared thermal imaging.	Patients with diabetic neuropathy had higher MFT and longer duration of diabetes compared to those without neuropathy, with a positive correlation for MFT and VPT on the big toe. In contrast, HbA1c levels showed no correlation with MFT values.	Thermal infrared imaging may serve as a valuable complementary tool for the high-risk assessment of diabetic feet.
4	Infrared thermal imaging for automated detection of diabetic foot complications [34]	van Netten JJ, van Baal JG, Liu C, van der Heijden F, Bus SA	2013	A Pilot Study	15 participants with diabetes	Investigate the potential of high-resolution thermal infrared imaging to non-invasively and automatically detect symptoms of diabetic foot disease and distinguish between various levels of complications.	The thermal infrared imaging showed that patients with localised foot complications had a temperature ROI more than 2 °C higher than the corresponding area on the contralateral foot and the mean temperature of the ipsilateral foot. In contrast, those with diffuse foot complications had an ROI with a temperature difference of more than 3 °C compared to the contralateral foot.	Thermal infrared imaging shows potential for non-invasive, automated detection of diabetic foot complications, but further research is needed to confirm these initial findings.
5	Morphological pattern classification system for plantar thermography of patients with diabetes [35]	Mori T, Nagase T, Takehara K, et al.	2013	The Study	161 participants (32 in the control group and 129 with diabetes)	Established a new objective thermographic classification system to identify and assess forefoot temperature patterns in patients with diabetes more accurately compared to the previous subjective method.	In examining foot thermographic patterns, those without diabetes showed four main categories with 17 anomalous patterns. In contrast, patients with diabetes showed six groups with significant abnormal patterns, especially types 6 and 7, and those with higher temperature patterns (types 2 and 4) had better circulation indices (ABI and TBI).	A new computerised classification system for sole thermography patterns reveals objectively more significant variability in patients with diabetes compared to non-diabetic controls. It aims to improve the daily foot care assessment.
6	Automatic classification of thermal patterns in diabetic foot based on morphological pattern spectrum [36]	D. Hernandez-Contreras, H. Peregrina-Barreto, J. Rangel-Magdaleno, J. Ramirez-Cortes, F. Renero-Carrillo	2015	The Study	60 participants (30 in the control group and 30 with diabetes)	Developed an innovative method to characterise and identify temperature patterns in thermographic images of the feet to aid in the early diagnosis and monitoring of patients with diabetes.	A neural network classifier was developed to distinguish temperature patterns in thermograms of diabetic patients and a control group.	The study presents a method to classify temperature patterns in healthy and diabetic patients using a neural network with the Levenberg-Marquardt algorithm. The highest classification accuracy, up to 94.33%, was achieved by combining pattern spectrum and positional data at low computational cost.
7	Automatic Analysis of Plantar Foot Thermal Images in at-Risk Type II Diabetes by Using an Infrared Camera [37]	Vilcahuaman L, Harba R, Canals R, et al.	2015	The Study	82 participants with diabetes	Developed and validated an automatic method using infrared imaging to analyse changes in the sole temperature of the foot in people with type II diabetes to identify early signs of foot ulceration and assess the risk of ulcer development.	The TOD differs significantly between the intermediate and high-risk groups. The sole temperature of the foot increases by one °C from moderate to high risk, indicating that these factors may be crucial in the early detection of diabetic foot.	Thermography combined with automated image processing could help in the early detection and prevention of foot ulcers in patients with DM, with further development and testing of the technology planned for future clinical use.
8	Anthropometric profile and diabetic foot risk: a cross-sectional study using thermography [38]	Neves EB, Almeida AJ, Rosa C, Vilaca-Alves J, Reis VM, Mendes R.	2015	The Study	44 participants with diabetes	Assesses the association between BMI, body fat percentage, and foot temperature asymmetry as indicators of diabetic foot risk in patients with type 2 diabetes.	There was a significant positive association between BMI and body fat percentage and temperature asymmetry in the feet, identifying three participants with diabetes-related foot complications, all of whom were obese and had high levels of body fat.	Positive association between higher BMI and body fat percentages and an enhanced risk of diabetic foot in patients with type 2 diabetes.
9	Automatic detection of diabetic foot complications with infrared thermography by asymmetric analysis [39]	Liu C, van Netten JJ, van Baal JG, Bus SA, van der Heijden F.	2015	The Study	76 participants with diabetes	An intelligent telemedicine system using infrared thermography and advanced image registration technology was developed to automatically assess the risk of diabetic foot complications by detecting temperature variations between corresponding areas of opposite feet despite challenges such as segmentation errors and foot deformities.	Achieved foot segmentation on colour images using the CIE Lab* colour space and EM-LDC or EM-QDC approaches, and successful registration and detection of diabetic foot complications on thermal images, with high sensitivity and specificity, but noted limitations in detection of complications when one foot is amputated, or both feet have similar problems.	Thermal image analysis, combined with advanced segmentation and registration techniques, holds promise for the early detection and monitoring of diabetic foot complications, with the potential for future improvement through integration into other imaging modalities and telemedicine systems.
10	Use of smartphone-attached mobile thermography assessing subclinical inflammation: a pilot study [40]	Kanazawa T, Nakagami G, Goto T, et al.	2016	A Pilot Study	16 images.	Validate the reliability and validity of the FLIR ONE device connected to a smartphone to assess inflammation by relative temperature rise compared to standard thermography used to assess decubitus ulcers and the diabetic foot.	It demonstrated excellent agreement (kappa coefficient of 1.00) regarding criterion-related validity and interpersonal and intrapersonal reliability between FLIR ONE and a high-end handheld infrared thermography device.	FLIR ONE can be a reliable alternative for assessing subclinical inflammation in pressure sores and diabetic feet, potentially supporting its routine use in the clinical setting.
11	A quantitative index for classification of plantar thermal changes in the diabetic foot [41]	D. Hernandez-Contreras, H. Peregrina-Barreto, J. Rangel-Magdaleno, J.A. Gonzalez-Bernal, L. Altamirano-Robles	(2017).	Regular article	140 participants (40 with the control group and 100 with diabetes)	Developed a quantitative index to measure thermal changes in the soleus region in people with diabetes.	In DM, the TCI shows a range of changes, from slight to severe deviations from the regular butterfly pattern. As thermal changes progress, TCI values increase, indicating more severe changes in sole temperature, and a new classification system based on TCI values has been proposed to quantify these changes, with categories ranging from mild (grade 1, TCI ≤ 2) to severe (grade 5, TCI > 5).	Establishes a novel method to classify thermal changes in the sole distribution of diabetic feet using a TCI, offering a more objective and accurate assessment than visual interpretations. The proposed TCI-based classification effectively detects and measures significant thermal changes, thus providing valuable information for monitoring and preventing diabetic foot complications.
12	A medical thermal imaging device for the prevention of diabetic foot ulceration [42]	Machin G, Whittam A, Ainarkar S, et al.	2017	Study	103 healthy participants	A significant reduction in the incidence of diabetic foot ulcers (DFU) was observed using a thermal imaging device in a clinical trial, comparing standard care with the addition of thermal imaging in patients at high risk of DFU.	Healthy feet generally showed thermal symmetry, with whole-foot temperatures averaging 29.1 ± 2.4 °C. However, spot temperatures varied more, and in some cases, significant differences in foot temperature were associated with factors such as recent physical activity or ill-fitting footwear.	The thermal imaging device developed to detect the early signs of DFU was effective, user-friendly, and suitable for clinical use, with potential applications in other medical areas such as decubitus ulcers, reconstructive surgery, and thyroid disease.
13	Diabetic foot ulcer mobile detection system using smartphone thermal camera: a feasibility study [43]	Fraiwan L, AlKhodari M, Ninan J, Mustafa B, Saleh A, Ghazal M.	2017	Feasibility study	-	Developed and implemented a mobile thermal imaging system that can detect early signs of diabetic foot ulcers automatically by analysing temperature changes on thermal images.	Proposed techniques were tested on thermal images of feet with simulated ulcers, using Otsu thresholding and the point-to-point difference method, effectively identifying temperature anomalies. However, segmentation errors were noted in some cases.	The system offers a framework for a mobile tool to help diabetic patients self-check their foot ulcers. However, it stays indicative rather than diagnostic, with future updates focusing on improving image quality with advanced thermal imaging cameras.
14	Enhanced thermal imaging of wound tissue for better clinical decision-making [44]	Keenan E, Gethin G, Flynn L, Watterson D, O’Connor GM.	2017	A Pilot Study	14 participants (11 patients with non-infected foot ulcers and three non-diabetic wounds)	Investigated the variability in the emissivity of chronic wounds and its impact on thermal measurements to improve the accuracy of clinical assessments.	The digital and thermal imaging of diabetic and non-diabetic wounds showed that wounds with eschars had a lower temperature than intact skin, wounds without eschars had a higher temperature in the centre and a cooler temperature at the edges, and healing wounds showed a decrease in both emissivity and temperature over time.	Emissivity significantly affects the accuracy of thermal imaging of open wounds, and incorporating emissivity correction into standard protocols can improve clinical decision-making.
15	Is Thermal Imaging a Useful Predictor of the Healing Status of Diabetes-Related Foot Ulcers? A Pilot Study [45]	Aliahmad B, Tint AN, Poosapadi Arjunan S, et al.	2018	A Pilot Study	26 participants with plantar neuropathic DRFU (11 healing and 15 nonhealing cases)	The rate of DRFU within the first four weeks of ulcer onset, it was predicted that thermal imaging would be used to measure the ulcers’ area and temperature distribution.	In cases of wound healing, the wound bed area index measured by thermal imaging at two weeks was significantly lower and correlated with a 50% reduction in area at four weeks. In contrast, colour imaging measurements and wound bed temperature did not show such an association.	The thermography, using isothermal patches to measure the ulcer area, shows promising results in the early prediction of DRFU healing. However, further studies with larger sample sizes and extended follow-up periods must confirm these results.
16	Standard Protocol of Preparation of the Subject with Risk Foot for Taking Images by Infrared Thermography [46]	PicadoÁA, Elena EM and Beatriz GM	2018	Research article	479 participants (202 in the control group and 277 with diabetes)	Provide a standard product for fabricating devices with feet at risk for thermographic imaging in the radio range, generated by measurable and non-contact visualisation of surface temperature changes using high-performance thermal imaging cameras.	The controlled environmental factors and participant conditions were used to ensure accurate thermographic imaging of foot temperature, using standard protocols and advanced equipment to detect diabetic foot complications through the analysis of thermal asymmetry.	Demonstrates that infrared thermography, as a safe, non-invasive, and cost-effective technique, enables accurate measurement and monitoring of critical parameters necessary for early detection of diabetic foot symptoms by rapid and non-contact recording of body radiation energy.
17	Reliability of a novel thermal imaging system for temperature assessment of healthy feet [47]	Petrova NL, Whittam A, MacDonald A, et al.	2018	The Study	105 participants	Demonstrated that infrared thermography, as a safe, non-invasive, and cost-effective technique, enables accurate measurement and monitoring of critical parameters necessary for early detection of diabetic foot symptoms by rapid and non-contact recording of body radiation energy.	The novel thermal imaging device demonstrated near-perfect agreement with a handheld infrared thermometer when assessing foot temperature in five regions of interest. It revealed its reliability and potential advantages for comprehensive foot temperature measurement.	It developed a newly developed thermal imaging device that is accurate, reliable and repeatable, making it a valuable tool for identifying patients at risk of developing diabetic foot ulcers in a clinical setting.
18	Mobile Application for Ulcer Detection [48]	Fraiwan L, Ninan J, Al-Khodari M.	2018	Feasibility study	-	It developed and implemented a mobile application that uses imaging to assess the presence of diabetic ulcers by using differential temperature between feet, allowing for early and accurate detection of the disease.	The algorithm was used on four images using OpenCV software to successfully identify and highlight ulcers in BGR images at a MTD of 2.2 °C, with variable MTD values ​​ranging from 1.8 °C to 2.5 °C in the simulated ulcer components.	The FLIR ONE IRT ulcer detection smartphone app offers a portable, user-friendly solution for early diagnosis of diabetic foot ulcers, with the potential for further improvement by integrating built-in smartphone thermal cameras.
19	The Application of Medical Thermography to Discriminate Neuroischemic Toe Ulceration in the Diabetic Foot [49]	Gatt A, Falzon O, Cassar K, et al.	2018	A Pilot Study	12 participants with diabetes	It evaluated whether thermal imaging could help distinguish healthy feet, non-ulcerative feet, and neuroischemic feet with digital ulcers in patients with type 2 diabetes.	There are significant temperature differences between healthy type 2 diabetic feet and both neuro-ischemic groups. Still, no significant differences were observed between neuro-ischemic feet with and without ulcerations or between toes of the same foot with and without ulcerations.	The temperatures were significantly higher in feet affected by cerebral ischemia than in healthy feet, and infrared thermography can help identify feet at risk of complications. Still, there was no difference in temperature between ulcerated and non-ulcerated toes on the same foot.
20	Monitoring of pH and temperature of neuropathic diabetic and nondiabetic foot ulcers for 12 weeks: An observational study [50]	Gethin G, O’Connor GM, Abedin J, et al.	2018	A Pilot Study	50 participants (34 with diabetes)	Assessed the surface pH, size, and surface temperature of uninfected neuropathic ulcerative lesions at baseline and after 12 uses in a cohort of patients.	The beginning showed an average pH of the wounds of 6.95, temperature of 30.91 °C and size of 0.82 cm². After 12 weeks, 50% of the wounds were healed, the average pH was 6.72, the temperature was 30.88 °C, and the size was 0.13 cm².	Demonstration of the utility of baseline pH and temperature in noninfected neuropathic foot ulcers. However, further studies in larger groups are needed to assess whether these biomarkers can reliably indicate healing or nonhealing states.
21	Plantar Thermogram Database for the Study of Diabetic Foot Complications [51]	Daniel Hernández-Contreras, Hayde Peregrina-Barreto, Jose Rangel-Magdaleno, Francisco Renero-Carrillo	2019	The Study	167 participants (45 with the control group and 122 with diabetes)	Demonstrates the introduction of a new public database of sole thermograms to facilitate research into the use of infrared thermography for the early diagnosis of diabetic foot complications, while detailing the acquisition protocol and reviewing appropriate image processing techniques.	The developed preparation, processing, and analysis of sole thermograms that were meticulously carried out, including patient acclimatisation, foot segmentation, and angioma extraction, resulted in a comprehensive database of 334 thermograms used to calculate temperature indices and assess diabetic foot status.	The database of sole thermograms facilitates early detection of diabetic foot complications by enabling analysis of vascular temperature changes while highlighting unresolved issues regarding segmentation, registration, and foot posture correction techniques.
22	Comparative thermal map of the foot between patients with and without diabetes through the use of infrared thermography [52]	Astasio-Picado Á, Escamilla Martínez E, Gómez-Martín B.	2019	Brief original article	479 participants (202 in the control group and 277 with diabetes)	The analysis of foot temperature variation in patients with and without diabetes using infrared thermography by segmenting the sole into four study areas.	The participants with diabetes had consistently higher foot temperatures than those without, indicating a significant temperature difference between the two groups.	Infrared Thermography can effectively assess temperature changes in feet at risk, helping to diagnose and prevent foot injuries in healthcare.
23	Validation of low-cost smartphone-based thermal camera for diabetic foot assessment. [53]	van Doremalen RFM, van Netten JJ, van Baal JG, Vollenbroek-Hutten MMR, van der Heijden F.	2019	A Pilot Study	32 participants with diabetes	It demonstrates a smartphone-based infrared camera with near-perfect compliance and high diagnostic accuracy compared to a high-end infrared camera for assessing diabetic foot ulcers.	The plantar and regional foot skin temperatures in participants with peripheral neuropathy showed excellent reliability and reasonable agreement in measurements, with minimal exclusions from the analysis.	The smartphone with a thermal infrared camera is a reliable and credible tool for assessing temperature differences in diabetic feet, making it useful for monitoring and preventing diabetic foot ulcers in clinical practice.
24	Comparison of Thermal Foot Maps between Diabetic Patients with Neuropathic, Vascular, Neurovascular, and No Complications. [54]	Astasio-Picado Á, Martínez EE, Gómez-Martín B.	2019	The Study	277 participants with diabetes	The use of infrared thermography to analyse temperature differences in the soles of diabetic patients’ feet, segmented into four areas, to identify neuropathy, vasculopathy, neurovascular disease, or the absence of these conditions.	The patients with neuropathy, vasculopathy, and neurovasculopathy showed lower temperatures in specific foot areas than those without these pathologies.	This demonstrates that Infrared Thermography is a valuable tool for assessing the risk of diabetic foot by detecting temperature changes that can help identify potential lesions in at-risk areas of the foot.
25	Between-visit variability of thermal imaging of feet in people attending podiatric clinics with diabetic neuropathy at high risk of developing foot ulcers [55]	Macdonald A, Petrova N, Ainarker S, et al.	2019	A Pilot Study	96 participants with diabetes	It demonstrates the quantification of thermal changes in diabetic feet at high risk of ulceration to assess the potential of using thermal imaging for early detection and prevention of foot ulcers.	The intrapatient and interpatient temperature variations in diabetic neuropathy patients are comparable, with significant asymmetry and variability between contralateral foot regions, and a substantial proportion of patients exceeded temperature thresholds associated with imminent foot ulcers.	Numerous hotspots were identified on the feet of high-risk diabetic patients, but the clinical significance of these hotspots and their variability over time remains unclear.
26	Infrared thermography and ulcer prevention in the high-risk diabetic foot: data from a single-blind multicentre controlled clinical trial [56]	Petrova NL, Donaldson NK, Tang W, et al.	2020	The Study	110 participants (61 in the control group and 49 with diabetes)	They develop the usefulness of monthly thermography and standard foot care to reduce diabetic foot ulcer recurrence.	The intervention group did not show a statistically significant reduction in ulcer recurrence compared to the control group in multiple analyses, with odds ratios ranging from 0.55 to 0.82 and risk ratios from 0.67 to 0.84, all with P-values greater than 0.22.	The monthly thermal imaging interventions did not significantly reduce ulcer recurrence or increase ulcer-free survival. Still, the study directed the design of a more sophisticated survey with extended follow-up and stratification of groups to better assess the effectiveness of thermography.
27	Deep Learning Classification for Diabetic Foot Thermograms [57]	Cruz-Vega I, Hernandez-Contreras D, Peregrina-Barreto H, Rangel-Magdaleno JJ, Ramirez-Cortes JM.	2020	The Study	110 participants with diabetes [Database: Hernandez-Contreras, D.A.; Peregrina-Barreto, H.; de Jesus Rangel-Magdaleno, J.; Renero-Carrillo, F.J. Plantar Thermogram Database for the Study of Diabetic Foot Complications. IEEE Access 2019, 7, 161296–161307,]	The analysis and comparison of the effectiveness of AI and DL techniques, including transfer learning models such as AlexNet and GoogleNet, as well as a newly designed DL framework, for classifying diabetic foot thermograms to improve the detection of abnormal sole temperature changes that may indicate a higher risk of ulceration.	The DE for automatic segmentation optimises fuzzy entropy for efficient ROI extraction. Among the classifiers tested, deep learning models, particularly custom DFTNet, outperformed ANN and SVM in classifying diabetic foot thermograms, although challenges remained with similar neighbouring classes.	The traditional classifiers, such as ANN and SVM, provided satisfactory results after feature extraction; the proposed DFTNet CNN project achieved excellent performance in classifying thermal images of diabetic patients, offering a promising direction for future improvements in automatic ulcer prediction with minimal expert intervention.
28	Use of a Smartphone Thermometer to monitor Thermal Conductivity Changes in diabetic foot ulcers: a pilot study [58]	Maddah E, Beigzadeh B.	2020	A Pilot Study	7 participants with diabetes	The reliability of a smartphone-connected thermal device in measuring temperature differences between damaged and adjacent tissues in diabetic foot ulcer patients is evaluated. It assesses the potential for using thermal conductivity changes as an early detection method for DFU.	The temperature variations and thermal conductivity reductions in diabetic foot wounds were significant. Patient 4 showed the highest temperature difference (6.9 °C) and the most significant decrease in thermal conductivity (84.3%), while Patients 5 and 6 exhibited lower but notable changes.	The smartphone-based FLIR ONE infrared thermometer is a reliable tool for diagnosing wound symptoms, inflammation, and metabolic and microvascular disorders in diabetic patients. It suggests using bright thermal sheets on vulnerable areas to prevent complications like vascular issues and gangrene.
29	Infrared 3D Thermography for Inflammation Detection in Diabetic Foot Disease: A Proof of Concept [59]	van Doremalen RFM, van Netten JJ, van Baal JG, Vollenbroek-Hutten MMR, van der Heijden F.	2020	A Pilot Study	8 participants with diabetes	The validity of the concept of 3D thermal foot imaging, combining 3D models with thermal imaging to improve the detection of diabetic foot ulcers beyond traditional 2D methods.	The 3D thermography results of eight participants with peripheral neuropathy and diabetic foot ulcers were successfully processed, revealing thermal anomalies despite minor technical errors and coverage limitations, and face reliability was rated positive in most cases.	This demonstrates a viable method for creating 3D thermograms to detect inflammation in diabetic foot disease, paving the way for improved image processing techniques and broader clinical applications.
30	Plantar temperature and vibration perception in patients with diabetes: a cross-sectional study [60]	Bhargavi A., Anantha K., Janarthan K.	2020	The Study	100 participants (50 in the control group and 50 with diabetes)	The effectiveness of combining thermal foot images and vibration perception data in early detecting pre-ulcerative lesions in diabetic feet will be evaluated.	Combining thermal imaging data and vibration perception led to fewer false alarms than models based on a single variable.	This indicates that multi-parameter data increases confidence in classifying factors that lead to pre-ulcerative changes in diabetic feet more effectively than relying on a predictive model with a single variable.
31	A machine learning model for the early detection of diabetic foot. thermogram images [61]	Amith Khandakar, Muhammad E.H. Chowdhury, Mamun Bin Ibne Reaz, Sawal Hamid Md Ali, Md Anwarul Hasan, Serkan Kiranyaz, Tawsifur Rahman, Rashad Alfkey, Ahmad Ashrif A. Bakar, Rayaz A. Malik	(2021).	Regular article	167 participants (45 control group and 122 with diabetes) [Database: D. A. Hernandez-Contreras, H. Peregrina-Barreto, J. d. J. Rangel-Magdaleno and F. J. Renero-Carrillo, “Plantar Thermogram Database for the Study of Diabetic Foot Complications,” in IEEE Access, vol. 7, pp. 161296–161307, 2019, doi: 10.1109/ACCESS.2019.2951356. keywords: {Foot; Diabetes; Image segmentation; Databases; Temperature distribution; Shape; Image edge detection; Diabetes mellitus; diabetic foot; infrared thermography; thermogram database},]	They develop a robust machine-learning-based solution using images of foot thermograms to identify diabetic foot ulcers, to compare the shallow CNN model and the AdaBoost classifier, and to demonstrate its potential for implementation as a smartphone app for home monitoring of diabetic foot ulcer progression.	The AdaBoost classifier with Random Forest feature selection outperforms deep CNN models in detecting diabetic foot, achieving a sensitivity of 96.71% using optimised feature sets. In contrast, Gamma-enhanced dual-foot thermograms significantly improve classification performance compared to single-foot thermograms.	The machine learning-based framework for early detection of diabetic foot ulcers from thermogram images offers a promising, easily deployable smartphone solution that outperforms traditional deep learning methods, potentially improving patient outcomes and reducing the burden on healthcare systems.
32	Assessment of Registration Methods for Thermal Infrared and Visible Images for Diabetic Foot Monitoring [62]	González-Pérez S, Perea Ström D, Arteaga-Marrero N, et al.	2021	Feasibility study	-	They analysed and evaluated the performance of four different recording methods to align thermal infrared and visible images captured by an inexpensive, camera-based prototype for remote monitoring of the diabetic foot, using several overlapping benchmark tests and testing their robustness at different distances.	All four image registration methods (GOT, Homography, ICP and Affine-ASGD) performed well at the focal plane distance (800 mm), with Affine-ASGD showing the best overall performance; their effectiveness decreased as the distance from the sensors increased, especially when the subject moved away from the focal plane.	The registration methods evaluated are suitable for aligning thermal infrared and visual footage, with a Dice factor above 0.950. However, keeping the working distance close to the focal plane is crucial for optimal accuracy.
33	Thermographic Characteristics of the Diabetic Foot With Peripheral Arterial Disease Using the Angiosome Concept [63]	Carabott M, Formosa C, Mizzi A, Papanas N, Gatt A.	2021	The Study	42 limbs	The temperature changes in the forefoot vasculature after limb elevation in type 2 diabetic patients with and without peripheral arterial disease (PAD).	The patients with PAD had a higher initial forefoot temperature than those without PAD, with significant differences in the medial and lateral forefoot. However, limb elevation only substantially affected the lateral forefoot after 1 min.	The patients with PAD show a higher forefoot temperature, but the foot elevation did not significantly change the thermal pattern.
34	Early diagnosis of diabetic peripheral neuropathy based on infrared thermal imaging technology [64]	Zhou Q, Qian Z, Wu J, Liu J, Ren L, Ren L.	2021	The Study	120 participants (60 in the control group and 60 with mild DPN)	They detect and compare the surface temperature of the soleus vessels in patients with mild diabetic peripheral neuropathy (DPN) and healthy control subjects to investigate a simple, convenient method for early detection of DPN diagnosis and assessment of the impact of sex and age on vascular surface temperature.	The skin temperature measurements were reliable, and there were significant differences in surface temperature between patients with mild DPN and healthy control subjects, except for the posterior tibial artery, with no discernible effect of gender or age.	The infrared detection of lower limb arterial surface temperature can reveal early abnormalities in patients with mild DPN, offering a non-invasive, practical approach to early diagnosis and understanding the relationship between body surface temperature and haemodynamic parameters.
35	Prevention of diabetic foot ulcers using a smartphone and mobile thermography: a case study [65]	Oe M, Tsuruoka K, Ohashi Y, et al.	2021	A case study	2 participants with diabetic neuropathy	The effectiveness of a smartphone-connected thermograph for self-monitoring foot temperature to prevent diabetic foot ulcers (DFU) in patients with diabetic neuropathy will be evaluated.	The device successfully enabled one patient to recognise an elevated foot temperature as a sign of potential inflammation. Still, the other patient showed no detectable temperature change during the testing period.	The device shows promise in promoting self-care to prevent DFU, but improvements are needed to detect high-risk thermographic lesions automatically.
36	A Deep Learning Method for Early Detection of Diabetic Foot Using Decision Fusion and Thermal Images [66]	Khairul Munadi, Khairun Saddami, Maulisa Oktiana, Roslidar Roslidar, Kahlil Muchtar Melinda Melinda, Rusdha Muharar, Maimun Syukri, Taufik Fuadi Abidin and Fitri Arnia	2022	Regular article	187 participants (45 control group and 142 with diabetes)[DatabaseHernandez-Contreras, D.A.; Peregrina-Barreto, H.; de Jesus Rangel-Magdaleno, J.; Renero-Carrillo, F.J. Plantar thermogram Database for the study of diabetic foot complications. IEEE Access 2019, 7, 161296–161307]	They develop and evaluate a framework for the early detection and classification of diabetic feet using thermal imaging and advanced fusion models based on pre-trained CNNs.	The simulation results of decision fusion models, particularly those combining MobileNetV2 and ShuffleNet, achieved 100% accuracy, sensitivity, specificity, precision and F-measure, outperforming single CNN models and traditional machine learning methods with fewer learning parameters and smaller model sizes.	The combines MobileNetV2 and ShuffleNet through decision fusion, achieves better performance metrics in classifying diabetic foot ulcers with smaller model size and fewer parameters, and future research aims to increase the generalisability of the dataset and explore additional fusion strategies for compatibility with mobile devices.
37	Thermal Change Index-Based Diabetic Foot Thermogram Image Classification Using Machine Learning Techniques [67]	Khandakar A, Chowdhury MEH, Reaz MBI, et al.	2022	Article	[Database: Hernandez-Contreras, D.; Peregrina-Barreto, H.; Rangel-Magdaleno, J.; Gonzalez-Bernal, J.; Altamirano-Robles, L. A quantitative Index for classification of plantar thermal changes in the diabetic foot. Infrared Phys. Technol. 2017, 81, 242–249., Hernandez-Contreras, D.A.; Peregrina-Barreto, H.; de Jesus Rangel-Magdaleno, J.; Renero-Carrillo, F.J. Plantar thermogram Database for the study of diabetic foot complications. IEEE Access 2019, 7, 161296–161307.]	The sole thermogram images of the foot, determined from the TCI, are classified using machine learning methods to improve the early diagnosis of diabetic foot complications, focusing on achieving high accuracy in multi-class classification.	The MLP classifier combined with XGBoost feature selection using the best features (average of LPA and LCA) achieved the highest performance, with a 91.18% weighted F1 score. In contrast, 2D CNN models with image enhancement techniques showed limited performance.	The machine learning framework excels in classifying diabetic foot thermograms into TCI-based classes, demonstrating the potential for remote healthcare applications. However, further validation with the new dataset is needed.
38	Segmentation of Plantar Foot Thermal Images Using Prior Information [68]	Bougrine A, Harba R, Canals R, Ledee R, Jabloun M, Villeneuve A.	2022	The Study	25 healthy participants	This develops a decision-support tool for preventing diabetic foot ulceration by introducing a new method for segmenting thermal images of the sole foot to accurately detect areas of hyperthermia as early signs of ulceration to integrate this method into a smartphone-based mobile analysis system for diabetic foot patients.	The s blind-segmentation methods struggled to segment thermal images of the soleus foot accurately due to noise. Still, snake segmentation methods based on prior shapes, particularly the proposed method, showed potential in achieving tighter alignment with truth contours, and quantitative metrics confirmed their superiority.	The proposed prior shape snake segmentation method for plantar foot thermal images showed excellent accuracy and fast convergence compared with existing methods. However, further refinement is required regarding parameter selection and handling of severely amputated feet, with ongoing efforts to enhance their applicability and expand their database.
39	Holistic multi-class classification & grading of diabetic foot ulcerations from plantar thermal images using deep learning [69]	Muralidhara S, Lucieri A, Dengel A, Ahmed S.	2022	The Study	167 participants (45 control group and 122 with diabetes) [Database: Hernández-Contreras D, Peregrina-Barreto H, Rangel-Magdaleno J, Renero-Carrillo F. Plantar thermogram database for the study of diabetic foot complications. IEEE Dataport. 2019. 10.21227/tm4t-9n15]	This developed and evaluated a novel convolutional neural network for accurate multiclass classification and severity grading of diabetic feet from plantar thermal images, outperforming previous methods and solving the problems of data imbalance and result variability.	The model achieved exceptional performance in detecting and classifying diabetic foot, with an average accuracy of 0.9827, an average sensitivity of 0.9684 and an average specificity of 0.9892.	The new benchmark for detecting and classifying diabetic foot ulcers through comprehensive multiclass classification of plantar thermograms, achieving high accuracy and sensitivity, and suggesting directions for future research, including precise ulcer prediction and integration with clinical data.
40	Development of a self-monitoring tool for diabetic foot prevention using smartphone-based thermography: Plantar thermal pattern changes and usability in the home environment [70]	Qin Q, Nakagami G, Ohashi Y, Dai M, Sanada H, Oe M.	2022	A Pilot Study	10 healthy participants	The feasibility of smartphone-based thermography for self-assessment of plantar thermal patterns at home will be assessed by analysing thermal images taken by healthy volunteers under different environmental conditions.	Survey results from the usability study revealed issues with the selfie stick and extension rod, and participants suggested improvements such as Bluetooth control, a tripod, a fixed camera viewing angle, daily measurements, an alarm reminder, and a pre-shot check to improve grip and ease of use.	The smartphone-based thermography is a practical and valuable self-assessment tool for home foot care in people with diabetes; to obtain reliable results, it is recommended that morning assessments be conducted and that health care professionals be communicated regularly.
41	Development of an AI classification model for angioma-wise interpretive substantiation of plantar feet thermal asymmetry in type 2 diabetic subjects using infrared thermograms [71]	Evangeline N C, Srinivasan S, Suresh E.	2022	The Study	153 participants with diabetes	The study classifies symmetric and asymmetric areas of the feet of diabetic patients using minimal colour image features. It evaluates the identified asymmetric areas to detect hotspots, indicating the onset of diabetic foot ulcers. The results demonstrate high accuracy and generalizability, as validated by machine learning.	Using a random forest model, classifying angioma regions into symmetric or asymmetric based on thermal profiles achieved high performance with a test accuracy of 96.07% and an F1 score of 0.96, and the model successfully identified hotspots indicating the risk of ulceration in people with diabetes with an accuracy of 92.5% in an independent public dataset.	They developed an AI model that accurately differentiates between symmetric and asymmetric areas in thermal images of the diabetic foot using minimal features. It achieved high accuracy and specificity and demonstrated its effectiveness in identifying ulcer-prone areas by analysing local temperature differences.
42	A Novel Machine Learning Approach for Severity Classification of Diabetic Foot Complications Using Thermogram Images [72]	Khandakar A, Chowdhury MEH, Reaz MBI, et al.	2022	The Study	167 participants (45 with the control group and 122 with diabetes)	The severity of diabetic foot complications is classified based on plantar foot thermogram images using a machine learning approach combining classical algorithms and convolutional neural networks to achieve high accuracy and reliability of early detection.	They used k-means clustering, classical machine learning, and two-dimensional CNN-based classification methods to effectively classify diabetic foot thermogram images into severity levels, achieving high accuracy and clinical significance.	The machine learning-based diabetic foot staging system using infrared thermograms can be effectively implemented as a web application, enabling remote healthcare using a simple infrared camera and a mobile app.
43	Early detection of diabetic foot ulcers from thermal images using the bag of features technique [73]	Mohammad H. Alshayeji, Silpa ChandraBhasi Sindhu, Sa’ed Abed	2023	The Study	167 participants (45 control group and 122 with diabetes) [Database: https://ieee-dataport.org/open-access/plantar-thermogram-database-study-diabetic-foot-complications]	They developed a highly accurate, real-time machine learning model for DFUs using thermal imaging, SIFT, SURF, and BOF techniques.	The SURF features combined with the SVM classifier achieved the best classification performance in distinguishing DFU thermal images from regular ones. At the same time, EfficientNetB0 features with SVM also showed promising results in this comparison.	A comprehensive machine learning model using SIFT and SURF techniques based on plantar thermogram images provides high accuracy in the early detection of DFUS, increasing the efficiency of diagnosis and potentially reducing hospital visits.
44	Region-wise severity analysis of diabetic plantar foot thermograms [74]	Sharma N, Mirza S, Rastogi A, Singh S, Mahapatra PK.	2023	The Study	104 participants (33 with the control group and 71 with diabetes)	Developed a method for early detection and severity classification of diabetic foot ulcers by analysing thermal images of the plantar foot using both conventional machine learning and deep learning techniques.	The CNN models, particularly InceptionV3, performed significantly better than CML models in classifying the severity of plantar foot ulcers, as evidenced by higher AUC scores in ROC analysis.	The CNN-based deep learning models significantly outperformed conventional methods for predicting the severity of diabetic foot ulcers using a new thermal dataset, suggesting that expanding the study group could further improve the accuracy of predictions and clinical assessment.
45	Definition of thermal indicators for the study of thermoregulation alterations in the foot of people living with diabetic peripheral neuropathy: A proof of concept [75]	Serantoni V, Jourdan F, Louche H, Avignon A, Sultan A.	2023	The Study	33 participants with diabetes	Investigate the relationship between diabetic peripheral neuropathy and impaired thermoregulatory mechanisms using thermal imaging, heat spectral analysis, and physical tests.	Thermal imaging analysis showed that exercise significantly increased plantar foot temperature, particularly in participants with lower initial foot temperature, and identified the mean wavelet in the neurogenic range as the most effective thermal index to distinguish individuals with peripheral neuropathy from those without.	The peripheral neuropathy in diabetic patients is associated with impaired foot thermoregulation. Although the study suggests that thermal imaging may improve neuropathy assessment, further studies with larger samples are needed to confirm these findings for clinical use.
46	Diabetic Plantar Foot Segmentation in Active Thermography Using a Two-Stage Adaptive Gamma Transform and a Deep Neural Network [76]	Cao Z, Zeng Z, Xie J, et al.	2023	A Pilot Study	22 participants (12 in the control group and 10 with diabetes)	They developed new methods, including a two-stage adaptive gamma transform and a PFSNet, to improve plantar foot segmentation in thermographic images of diabetic feet exposed to cold without relying on colour or depth information.	The PFSNet model outperforms five other deep learning models, including TransUNet, for plantar foot thermogram segmentation with the highest accuracy while being more efficient in size and training time.	The two-stage adaptive gamma transform and the PFSNet network demonstrated higher accuracy and efficiency for plantar foot segmentation from thermal images under thermal stress, outperforming existing deep neural networks, including TransUNet, due to smaller model sizes and lower computational costs.
47	Evaluation of the surface temperature distribution in the feet of patients with type 2 diabetes using thermal imaging method [77]	Dębiec-Bąk A, Skrzek A, Ptak A, Majerski K, Uiberlayová I, Stefańska M.	2023	The Study	85 participants (33 with the control group and 52 with diabetes)	To evaluate the distribution of surface temperature and microcirculation in the feet of patients with DM and to assess the potential of thermal imaging as a diagnostic tool for DFS.	The patients with type 2 diabetes had significantly higher mean foot surface temperature than the control group, with more significant temperature increases observed on the plantar and dorsal surfaces of the feet, indicating impaired microcirculation and an increased risk of DFS.	Thermal imaging can be a valuable tool in monitoring and managing foot health in patients with DM, detecting early changes in foot surface temperature, potentially indicating microcirculation disorders, and helping to prevent neurotrophic changes.
48	Leveraging intelligent image processing techniques for early detection of foot ulcers using a deep-learning network [78]	Garima Verma	2024	The Study	1055 total images (543 are standard foot images and 512 images of abnormal feet of the patient) [Dataset: Alshayeji MH, Sindhu SC, Abed S. Early detection of diabetic foot Ulcers from thermal images using the bag of features technique. Biomed Signal Process Control 2023; 79: 104,143. DOI: 10.1016/j.bspc.2022.104143]	They detect foot ulcers in diabetic patients using thermal images and evaluate the performance of the proposed deep learning model (ResNet50 and EfficientNetB0) compared with existing studies after extending the dataset by Canny edge detection, watershed segmentation, and data augmentation.	The EfficientNetB0 model outperforms ResNet50 and other state-of-the-art models in terms of accuracy and F1 score, especially after applying edge detection and segmentation preprocessing, showing improved performance on sizeable thermal image datasets.	The diabetic foot ulcer prediction model combining deep features with manually designed features using edge detection and segmentation techniques achieved a high accuracy of 0.994 and outperformed current methods, with the potential to be improved by integrating IoT devices for real-time prediction.
49	Automated Prediction of Diabetes Mellitus using Infrared Thermal Foot Images: Recurrent Neural Network Approach [79]	Kumar G, Arora AS.	2024	The Study	50 participants in the diabetic (without neuropathic conditions) and non-diabetic groups	The automated system uses thermal images of feet and a recurrent neural network to predict diabetes accurately, potentially improving patient outcomes by enabling noninvasive and timely screening.	The RNN model outperformed the Lw-CNN model in terms of higher accuracy (97.14% versus 82.9%), higher precision, sensitivity, specificity, F1 score, and MCC, indicating its better performance in predicting DM, primarily by capturing temporal dependencies in the data.	The thermography combined with advanced models such as DFPM and CNN-LSTM has high accuracy and potential as a noninvasive tool for predicting and monitoring DM and its complications. However, further studies are needed for their clinical validation.

*DM *diabetes mellitus, *PPN *peripheral polyneuropathy, *MFT *mean foot temperature, *VPT *vibration perception threshold, *HbA1c *glycated hemoglobin, *ROI* region of interest, *ABI *The Ankle-Brachial Index, *TBI *The Toe Brachial Index, *TOD* time of diagnosis, *BMI *body mass index, *TCI *thermal change index, *DFU/DRFU *diabetic foot ulcers, *MTD *mean surface temperature difference,*DE *differential evolution, *DL *deep learning, *ANN *artificial neural networks, *SVM* support vector machine, *DFTNet CNN* novel convolutional neural network architecture, *CNN *convolutional neural network, *PAD *peripheral artery disease, *DPN* diabetic peripheral neuropathy, *MLP *multilayer perceptron, *SIFT* scale-invariant feature transform, *SURF *accelerated robust features, *BOF* bag-of-features, *ROC *receiver operating characteristic, *PFSNet* novel deep neural network, *DFS *diabetic foot syndrome

## Discussion

The discussion section has been divided into thematic subsections to ensure clarity and a systematic presentation of the results. This approach allows for an in-depth analysis of individual aspects of the issues studied and makes it easier for the reader to navigate the wide range of materials presented. The following topics are discussed in the rest of the paper: various methods and approaches used in research, the use of advanced automatic analysis and machine learning techniques, challenges related to the use of mobile thermal imaging cameras, and the relationship between patient characteristics and thermography results. There are also reflections on the possibility of predicting wound healing and indications of the main limitations of the current state of research.

## Overview of included studies and methodological approaches

Based on the collected articles, the usefulness of thermography as a diagnostic tool in detecting pre-ulcer conditions and complications such as ulcers in patients with diabetes was assessed. The articles presented a variety of approaches, including case studies [43, 48, 62] and the use of databases [51, 57, 61, 66, 67, 69, 72]. Although many studies included test and control groups, it was noted that these were often imbalanced in terms of numbers and characteristics [31, 35, 36, 41, 46, 50–52, 56, 60, 65, 66, 69, 72–77].

### Automated analysis and machine learning techniques

Several studies have used automatic analyses such as segmentation or neural networks [36, 37, 39, 47, 61, 69, 72]. The first such paper used automatic classification using a neural network based on the Levenberg-Marquardt algorithm, achieving 94.3% accuracy by combining analysis of temperature patterns with positional data [36]. Another study used convolutional neural networks (CNNs) for two-dimensional lesion classification, achieving high clinical performance [72]. Another study used the AdaBoost classifier with Random Forest feature selection in a survey based on foot thermograms, which attained a sensitivity of 96.71%, outperforming CNN models, and thermograms of both feet increased classification performance compared to single thermograms [67]. Another paper showed that the mean temperature of the sole surface of the feet in the high-risk group was 1 °C higher than in the medium-risk group [37]. A subsequent publication used a neural network to multi-classify and assess the severity of diabetic feet based on thermal images of the sole. The model achieved high accuracy (0.9827), sensitivity (0.9684), and specificity (0.9892), outperforming previous methods in ulcer detection and classification [69]. Each of these papers cited the possibility of using the models in the future, with the majority needing to expand the research group. The study by Liu et al. focused on thermal image segmentation and image registration of the diabetic foot using the CIE Lab* colour space, achieving high sensitivity and specificity. However, difficulties arose in cases of single-foot amputation or when both feet showed similar problems [39]. Other studies using deep learning and artificial intelligence, such as the DFTNet model, achieved better results than classical classifiers, such as ANN or SVM, using differential evolution to automatically segment images [47]. Each of these papers reported on the possibility of using the models in the future, but most need to enlarge the research group.

## Computational efficiency and resource requirements

The table below provides an overview of selected deep learning-based models, including their number of parameters, approximate prediction time, and hardware requirements (CPU/GPU). The data was estimated based on information available in the cited publications or based on known architecture configurations (Table 2).

**Table 2:** Comparison of selected machine learning models in terms of number of parameters, prediction time, and hardware requirements (CPU/GPU). Data was estimated based on the cited publications

Article/Model	Number of parameters	Prediction time (ms)	Hardware (GPU/CPU)
DFTNet (Cruz-Vega et al., 2020)	~ 2.1 M	18 ms	GPU
AdaBoost + RF (Khandakar et al., 2021)	N/A	5 ms	CPU
MobileNetV2 + ShuffleNet (Khairul Munadi et al., 2022)	3.4 M + 1.2 M	10–15 ms	GPU
EfficientNetB0 (Garima Verma, 2024)	5.3 M	20 ms	GPU
XAI-FusionNet (Biswas et al., 2024)	ok. 6–10 M	25 ms	GPU
MLP + XGBoost (Khandakar et al., 2022)	N/A	6 ms	CPU

The analysed studies used lightweight models (e.g., MobileNetV2, ShuffleNet [66]) and more complex architectures (e.g., DFTNet [57], EfficientNet [73]). Lightweight models are characterised by a smaller number of parameters (1–3 million), which results in shorter inference times (< 15 ms) and lower hardware requirements, allowing them to be used in mobile devices.

More complex models, such as EfficientNetB0 or DFTNet [57, 73], offer higher accuracy but require significantly more computing resources (GPU) and are larger (over 5 million parameters), which may limit their practical application.

In classical approaches, such as Ada [61], the computational complexity of Boost or MLP + XGBoost [67] is much lower, but they may achieve lower sensitivity and accuracy than neural networks. Their advantages remain in their speed and ease of implementation on CPUs.

For clinical and mobile applications, it is recommended to use lightweight models with limited complexity that can be integrated into mobile applications while maintaining an acceptable level of accuracy [66, 67].

## Computational complexity analysis

To assess the practical applicability of various machine learning models used in thermographic analysis of diabetic foot, the computational complexity of selected algorithms was evaluated based on model size, inference time, and hardware requirements.

Lightweight models such as MobileNetV2 and ShuffleNet [66] have relatively low parameter counts (approximately 1–3 million) and fast prediction times (below 15 ms), making them well-suited for mobile or edge computing environments.

In contrast, more complex architectures like EfficientNetB0 and DFTNet [57, 73] demonstrate higher diagnostic performance but require significantly more computational power, typically necessitating GPU-based inference and resulting in longer processing times.

Classical machine learning approaches offer much lower computational demands, such as AdaBoost [61] and MLP combined with XGBoost [67]. These models can operate efficiently on standard CPU-based systems, which makes them attractive for quick and lightweight deployment. However, they may provide slightly lower sensitivity and accuracy than deep learning-based frameworks.

In summary, selecting the most appropriate model for clinical or home-based applications requires balancing diagnostic accuracy with available computational resources. Lightweight convolutional neural networks [66, 72] represent a promising middle ground, offering sufficient performance with low resource consumption, and are suitable for mobile integration. In contrast, high-performance deep learning models [57, 73] are better suited for hospital-grade systems with dedicated hardware. Future work should also investigate energy efficiency, deployment scalability, and real-time performance for continuous monitoring applications.

### Mobile thermal imaging and standardization issues

Another topic is mobile thermal imaging cameras, such as FLIR ONE, which have the potential to self-monitor diabetic patients. However, lack of standardisation remains a problem in their use [40, 48, 53, 58, 65, 68, 70].

### Correlations between patient characteristics and thermographic findings

Studies also indicate a correlation between BMI and foot temperature asymmetry. Neves et al. showed a significant association between higher BMI and temperature asymmetry, particularly in patients with diabetes and foot complications [38]. In contrast, patients with diabetic neuropathy showed higher foot temperatures and longer duration of diabetes compared to those without neuropathy [31, 33].

### Thermographic patterns in localized foot complications

A thermographic study of patients with localised foot complications noted that the temperature in the affected areas was more than 2 °C higher than the opposite foot [34]. In contrast, healthy feet were characterised by thermal symmetry; however, the temperature differences could be due to recent physical activity or ill-fitting footwear [42].

### Thermography in predicting wound healing

Thermal imaging of wounds in predicting the healing process is another research topic. Keenan et al. noted that wounds without eschars had higher temperatures and that a decrease in temperature could signal clinical improvement [44]. Aliahmad et al. indicated that isothermal thermographic analysis could predict wound healing in as few as two weeks [45]. Gethin et al. highlighted the importance of monitoring pH and temperature in predicting healing. However, further larger-scale studies are needed [50].

### Limitations and challenges identified in current research

Thermography has excellent potential as a diagnostic tool in detecting and monitoring diabetic foot complications. Still, there is a need for standardisation of measurement methods and further research to confirm the effectiveness of these techniques on larger patient samples.

During the literature analysis and review of available studies on thermography in examining the diabetic foot, several significant limitations were identified that affect the results’ quality, reliability, and comparability.

One of the most common problems is the lack of reference to thermography results compared to the results of other, widely recognised diagnostic methods, such as laboratory tests. The lack of such a reference point significantly reduces the diagnostic value of thermography. It makes assessing its effectiveness as an independent tool in detecting and monitoring diseases difficult.

Another significant limitation is the quality of the equipment used. Many analyzed studies used low-resolution thermal imaging cameras, often intended for amateur use or working with smartphones. Although such devices are cheap and readily available, their limited precision and low sensitivity to subtle changes in body surface temperature can lead to incorrect interpretations in the medical context.

An additional problem is the lack of standardization of research protocols. The individual studies differed in measurement conditions (such as ambient temperature, patient acclimatization time, camera distance from the examined area), and methods of analysis and interpretation of the obtained thermographic images. Such heterogeneity makes it difficult to compare results between studies, and in many cases, even impossible. The lack of a standard, standardised measurement protocol also limits the possibility of creating larger, consistent data sets that could be used to train artificial intelligence algorithms supporting the diagnostic process.

### Future development directions

In the future, thermal imaging could become a fast, non-invasive, and reliable method for regular foot monitoring in patients with diabetes mellitus. With the help of advanced thermographic techniques, it will be possible to detect early changes in temperature distribution that may indicate the onset of inflammation, infection, or ulceration—even before clinical symptoms become visible.

Integration with AI-based diagnostic systems could also significantly enhance the accuracy and speed of analysis. Recent works have demonstrated that deep learning methods integrating multi-scale feature fusion and explainable AI (XAI) can dramatically improve DFU detection and clinical interpretability.

For instance, DFU_XAI proposed by Biswas et al. [80] utilizes Grad-CAM-based explainability to highlight critical regions in foot thermograms, enhancing trust and transparency in clinical environments. Similarly, the DFU_MultiNet model [81] achieves high sensitivity by fusing features at different scales, enabling robust ulcer detection even in thermograms with subtle differences.

Another significant contribution, XAI-FusionNet [82], further refines this approach by combining multi-scale fusion with XAI techniques, producing accurate and interpretable predictions. While these models focus on diabetic foot ulcers, their generalizability is also evident in other domains. The XEMLPD framework [83], developed for Parkinson’s disease diagnosis, demonstrates how optimized features and ensemble learning can offer explainable predictions in complex clinical contexts.

These advances collectively point toward the growing maturity of interpretable AI in medicine. As a result, future diagnostic tools based on thermal imaging could leverage these architectures to provide both actionable insights and visual explanations, supporting early intervention and improving clinician-patient communication.

Ultimately, these improvements may lead to the development of accessible, user-friendly tools for both clinical and home use, enabling earlier detection, better prevention strategies, and improved outcomes for patients with diabetic foot.

## Conclusion

Thermal imaging is a safe, non-invasive, and rapid diagnostic method that effectively supports the detection of early inflammation in people at elevated risk of developing diabetic foot. An analysis of the available articles highlights the crucial importance of correct examination methodology, including the correct division into test and control groups, which significantly affects the reliability and accuracy of the results obtained. Many authors have also highlighted the processing of thermograms and the equipment used to perform them. It is, therefore, essential to continue research to establish a uniform methodology for thermograms and analysis.

## Data Availability

No datasets were generated or analysed during the current study.
